# Pulmonary embolism as the initial manifestation of acute promyelocytic leukemia: A case report and review of the literature

**DOI:** 10.1097/MD.0000000000051334

**Published:** 2026-10-30

**Authors:** Jiayao Dong, Xiao Zhai

**Affiliations:** a Department of Hematology, The Eighth Affiliated Hospital of Southern Medical University (The First People’s Hospital of Shunde District, Foshan City), Foshan, Guangdong Province, China.

**Keywords:** acute promyelocytic leukemia, anticoagulation, case report, pulmonary embolism, thrombosis

## Abstract

**Rationale:**

Acute promyelocytic leukemia (APL) usually presents with bleeding-related coagulopathy. Pulmonary embolism (PE) before APL is recognized is uncommon and complicates anticoagulation.

**Patient concerns:**

A 26-year-old obese man presented with progressive dyspnea, small-volume hemoptysis, and pleuritic chest pain.

**Diagnoses:**

Computed tomography pulmonary angiography showed segmental PE in the right lower lobe. Leukocytosis, anemia, and severe thrombocytopenia prompted hematologic evaluation. Bone marrow morphology, t(15;17), and promyelocytic leukemia–retinoic acid receptor alpha confirmed high-risk APL.

**Interventions:**

Nadroparin was started for PE. After APL was confirmed, all-trans retinoic acid, hydroxyurea, pirarubicin, and later arsenic trioxide were administered. Anticoagulation was reduced during bleeding and thrombocytopenia and later resumed with transfusion support.

**Outcomes:**

Suspected differentiation syndrome resolved with dexamethasone. PE improved, but a splenic infarct developed during induction. Therapeutic anticoagulation was resumed after bleeding resolved. The patient achieved sustained molecular remission, complete radiologic resolution of PE, and no recurrent thrombosis during more than 2.5 years of follow-up.

**Lessons:**

PE with unexplained cytopenias or leukocytosis should prompt smear review and urgent evaluation for APL. Confirmed thrombosis requires anticoagulation individualized to thrombus severity, bleeding, platelet and fibrinogen trends, and serial reassessment.

## 1. Introduction

Acute promyelocytic leukemia (APL) is a genetically defined subtype of acute myeloid leukemia characterized in most patients by t(15;17)(q24;q21) and the promyelocytic leukemia–retinoic acid receptor alpha (PML::RARA) fusion. APL is a hematologic emergency because its coagulopathy can cause early death. The phenotype commonly combines consumptive coagulation, hyperfibrinolysis, and thrombocytopenia, making hemorrhage the dominant clinical concern. Current guidance therefore recommends initiating all-trans retinoic acid (ATRA) as soon as APL is suspected while aggressively supporting hemostasis.^[1]^

Thromboischemic events nevertheless form part of the same coagulopathy. In a 2026 registry analysis of 1210 patients with newly diagnosed APL, 195 (16%) developed a thromboischemic event; pulmonary embolism (PE) occurred in 2.1%, and most events arose at diagnosis or during induction.^[2]^ Reported incidence varies because studies use different event definitions and frequently include superficial or catheter-related thrombosis.^[2,3]^ PE that precedes recognition of APL remains particularly uncommon. Its management is difficult because therapeutic anticoagulation may be required while platelet counts and fibrinogen are falling and clinically relevant bleeding is evolving.

We report a young man in whom segmental PE was the first manifestation of high-risk APL and a splenic infarct subsequently occurred during induction. The aims are to clarify the diagnostic reasoning and treatment decisions, interpret the simultaneous thrombotic and hemorrhagic phenotypes, compare the case with previously reported presentations, and derive practical lessons for early recognition and individualized anticoagulation.

## 2. Materials and methods

### 2.1. Case report design

This retrospective single-patient report describes the diagnostic evaluation, treatment, and follow-up from May 2022 through early 2025. Clinical findings, laboratory and molecular results, treatments, and imaging findings are reported descriptively; no statistical analysis was performed. The report was de-identified and prepared in accordance with the CARE guideline.^[4]^

### 2.2. Focused literature review

PubMed was searched from database inception through August 14, 2026, using (“Leukemia, Promyelocytic, Acute”[MeSH] OR “acute promyelocytic leukemia”[All Fields] OR “acute promyelocytic leukaemia”[All Fields]) AND (“Pulmonary Embolism”[MeSH] OR “pulmonary embolism”[All Fields] OR “pulmonary emboli”[All Fields] OR “pulmonary thromboembolism”[All Fields] OR “pulmonary thromboemboli”[All Fields]). The query returned 22 records. Titles and abstracts were screened, then available full texts and the reference lists of eligible reports and APL-thrombosis reviews were examined because PE was not always mentioned in the title or abstract. Individual case reports were included when the authors explicitly reported imaging-supported PE before or contemporaneously with APL recognition. Treatment-emergent PE, cohort-only data, conference abstracts, reports without patient-level information, and reports in which PE or its timing could not be verified were excluded. Ten prior reports met these criteria and were summarized descriptively.^[5–14]^ This focused review was intended to contextualize the case; it was not registered or conducted as a systematic review.

### 2.3. Ethics and consent

Ethical review was not applicable to this retrospective single-patient report of routine clinical care because it involved no research intervention. Informed consent for publication was obtained from the patient.

### 2.4. Case presentation

#### 2.4.1. Patient information and clinical findings

A 26-year-old obese man with hypertension and hyperlipidemia was admitted to the intensive care unit on May 12, 2022, because of progressive dyspnea and hemoptysis. Six days before admission, he had developed a cough and rhinorrhea; dyspnea and small-volume hemoptysis followed 3 days later, and pleuritic chest pain developed 2 days before admission. He also reported fever that had not been objectively measured and night sweats. Breath sounds were diminished over the right lower lung field without rales. No skin ecchymosis, petechiae, lower-extremity edema, or cardiac murmur was present at the initial examination.

#### 2.4.2. Diagnostic assessment

Computed tomography pulmonary angiography obtained immediately before admission demonstrated acute emboli in the posterior and medial basal segmental arteries of the right lower lobe, together with bilateral pneumonia and a right pleural effusion (Fig. 1A1, A2). The white blood cell count was 24.51 × 10^9^/L, hemoglobin was 91 g/L, and the platelet count was 35 × 10^9^/L. D-dimer and fibrin degradation products were markedly elevated. Arterial blood gas analysis showed hypoxemia, with a ratio of arterial oxygen partial pressure to inspired oxygen fraction of 160 mm Hg. Bilateral lower-extremity venous ultrasonography showed no deep vein thrombosis.

**Figure 1.**
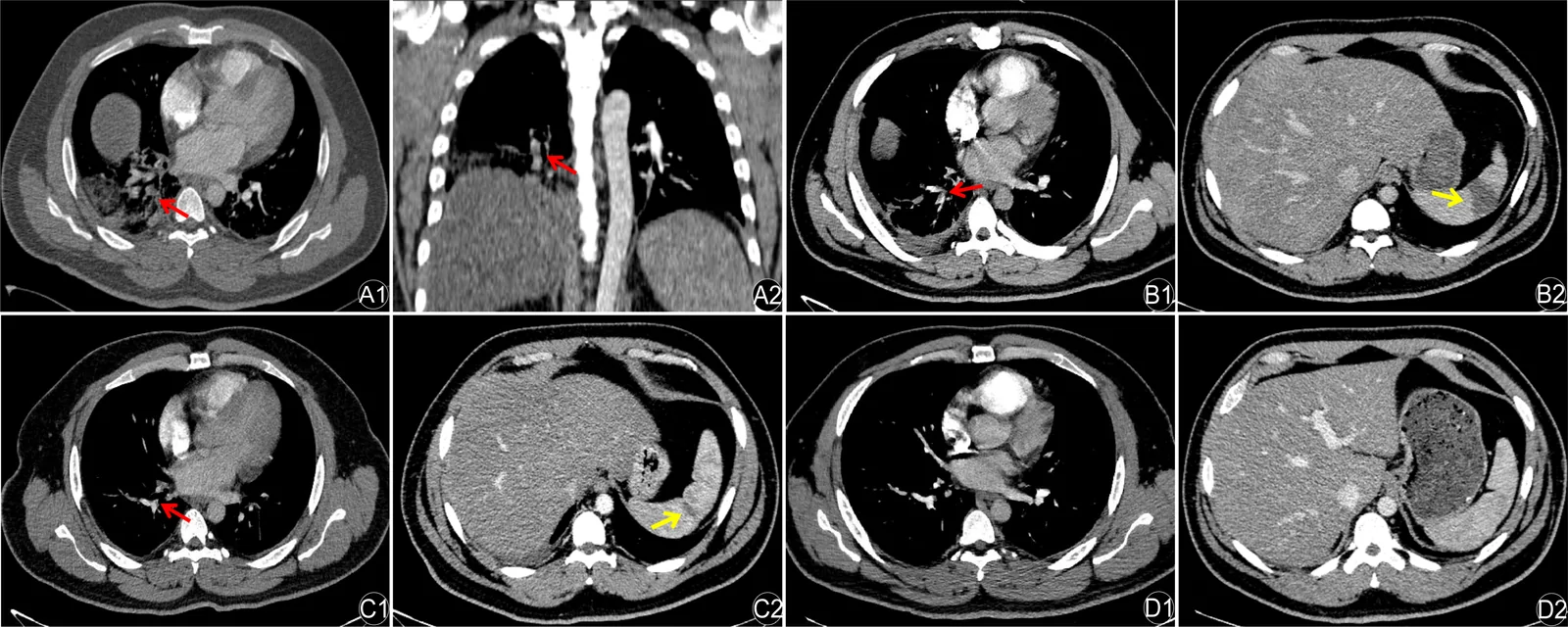
Serial computed tomography pulmonary angiography and abdominal imaging. (A1) and (A2) Initial study (May 11, 2022) showing segmental emboli in the right lower lobe. (B1) and (B2) Follow-up on June 7, 2022, showing improvement of pulmonary embolism and a new splenic infarct. (C1) and (C2) Follow-up in August 2022 showing chronic pulmonary embolic change and a stable splenic infarct. (D1) and (D2) Final study in early 2025 showing complete resolution of pulmonary embolism and chronic splenic-infarct scarring. Arrows identify the thromboembolic lesions.

The presenting combination of leukocytosis, anemia, and severe thrombocytopenia, together with subsequent progressive cytopenias and abnormal peripheral blood findings, raised suspicion of an underlying hematologic malignancy. Bone marrow examination on May 16 showed 90.1% abnormal promyelocytes. Cytogenetic testing identified t(15;17), and molecular testing detected PML::RARA, establishing APL. Because the presenting white blood cell count exceeded 10 × 10^9^/L, the disease met the European LeukemiaNet definition of high-risk APL.^[1]^

### 2.5. Therapeutic intervention and decision-making

Because symptomatic segmental PE had been objectively confirmed, nadroparin calcium was initiated at the reported regimen (1.0 mL subcutaneously every 12 hours), together with supplemental oxygen and empirical anti-infective therapy. A detailed chronology is provided in Table 1.

**Table 1:** Timeline of diagnostic, therapeutic, and outcome-defining events in the present case.

Date	Key milestone
May 11–12, 2022	Segmental right-lower-lobe PE was diagnosed; nadroparin was begun, and unexplained blood-count abnormalities prompted hematologic evaluation.
May 16	Bone marrow morphology, t(15;17), and PML::RARA confirmed high-risk APL; ATRA and cytoreduction were initiated, with anticoagulation attenuated as bleeding developed.
May 19–24	Pirarubicin and transfusion support were given; suspected differentiation syndrome on May 20 responded to dexamethasone, with stabilization documented on May 24.
May 29	ATO was added to ongoing ATRA.
June 7	CTPA showed improving PE and a new splenic infarct; therapeutic nadroparin was resumed after bleeding had resolved and hemostatic parameters improved.
June 11–30	Multidisciplinary review and marrow/molecular reassessment documented hematologic remission, followed by undetectable PML::RARA; consolidation was initiated.
June–October 2022	Anticoagulation was transitioned to rivaroxaban, withheld when platelets were <20 × 10^9^/L, and discontinued on October 1 after approximately 4.5 mo.
August 2022–early 2025	APL consolidation and maintenance were completed by June 2024; final imaging in early 2025 showed resolved PE and chronic splenic-infarct scarring, with sustained molecular remission.

APL = acute promyelocytic leukemia, ATO = arsenic trioxide, ATRA = all-trans retinoic acid, CTPA = computed tomography pulmonary angiography, PE = pulmonary embolism, PML::RARA = promyelocytic leukemia–retinoic acid receptor alpha.

After APL was confirmed, ATRA (20 mg orally twice daily) was started without delay. Hydroxyurea was used for leukocytosis control, and pirarubicin (20 mg intravenously on days 1 and 3) was added for cytoreduction. Arsenic trioxide was added to ongoing ATRA on May 29. As thrombocytopenia worsened and skin ecchymoses and conjunctival hemorrhage developed, nadroparin was temporarily reduced to 0.3 mL subcutaneously once daily. Platelet and plasma transfusions were administered.

Suspected differentiation syndrome responded promptly to dexamethasone.

### 2.6. Follow-up and outcomes

On June 7, computed tomography pulmonary angiography showed substantial improvement of the pulmonary emboli but also demonstrated a new splenic infarct (Fig. 1B1, B2). In the absence of active bleeding and with improving coagulation parameters, therapeutic nadroparin (1.0 mL subcutaneously every 12 hours) was resumed.

Bone marrow reassessment in June confirmed complete hematologic remission. PML::RARA was reported at 0.01% on June 11 and was undetectable on June 30. During consolidation, anticoagulation was transitioned to rivaroxaban and was withheld when the platelet count was below 20 × 10^9^/L. Anticoagulation was discontinued on October 1, 2022, after approximately 4.5 months. By then, the leukemia remained in molecular remission, and serial imaging had documented pulmonary improvement.

Serial imaging demonstrated progressive resolution of PE without recurrent thrombosis (Fig. 1C1–D2). APL-directed consolidation and maintenance therapy were completed, and molecular remission was sustained. At follow-up in early 2025 – more than 2.5 years after presentation – imaging showed complete resolution of PE and only chronic scarring from the splenic infarct.

## 3. Discussion

### 3.1. Bidirectional coagulopathy and interpretation of the index PE

The central observation is that objectively confirmed PE preceded APL-directed therapy; the index event therefore cannot be attributed to ATRA or chemotherapy. APL itself offers a biologically plausible prothrombotic substrate. Leukemic promyelocytes express tissue factor, and a human-subject study indicates that tissue factor-bearing microparticles transfer active tissue factor into plasma and contribute to coagulation activation.^[15]^ At the same time, consumption of coagulation factors, hyperfibrinolysis, and thrombocytopenia promote bleeding.^[1,3]^ These processes are not mutually exclusive, which explains why severe thrombocytopenia did not protect this patient from PE.

Causation remains multifactorial. Obesity and acute pulmonary infection may have contributed, while negative lower-extremity ultrasonography neither excludes a resolved venous source nor establishes in situ pulmonary thrombosis. Nevertheless, the abnormal blood counts were already present with PE, and a subsequent splenic infarct occurred while APL remained active. Together, these temporal features are consistent with a contribution from APL-associated coagulopathy, although the relative roles of APL, obesity, and infection cannot be separated in a single case.

The focused review identified 10 prior reports explicitly documenting PE before or contemporaneously with APL recognition.^[5–14]^ Presentations ranged from leukopenia to hyperleukocytosis and from preserved platelet counts to thrombocytopenia. This heterogeneity, also evident in contemporary cohorts,^[2,3]^ argues against relying on a single blood-count pattern to recognize thrombotic APL. The present case adds a young patient with severe thrombocytopenia, no ultrasonographic evidence of deep vein thrombosis, and sequential PE and splenic infarction.

### 3.2. Why thrombosis can persist during induction

The new splenic infarct occurred while pulmonary clot burden was improving, showing that the 2 vascular findings followed different trajectories during induction. At that time, active high-risk APL, leukocytosis, and cytoreduction, temporarily attenuated anticoagulation because of bleeding, and suspected differentiation syndrome coexisted. In vitro, ATRA increases adhesion of differentiating APL cells to endothelium,^[16]^ whereas differentiation syndrome is a recognized complication of ATRA-based induction^[17]^; neither observation establishes that treatment caused the splenic infarct. ATRA also treats the underlying leukemia and reduces its procoagulant drive. This case cannot separate disease-, treatment-, and patient-related contributions to the infarct.

### 3.3. Balancing anticoagulation and bleeding

Evidence for treating established thrombosis in APL is limited to observational data, case reports, and extrapolation from cancer-associated thrombosis. Guidance against routine heparin for APL coagulopathy does not address a separate indication such as objectively confirmed symptomatic PE.^[1]^ General cancer-associated thrombosis guidance considers symptomatic segmental or more proximal PE a high-risk event and supports therapeutic anticoagulation with platelet-transfusion support to maintain a platelet count of approximately 40 to 50 × 10^9^/L when severe thrombocytopenia is present; dose modification or interruption is considered when bleeding risk predominates.^[18]^ These thresholds are not validated specifically in APL and must be integrated with fibrinogen, active bleeding, clot burden, and the capacity for close monitoring.

In this patient, anticoagulation was reduced during overt bleeding and resumed at therapeutic intensity after bleeding resolved and hemostatic parameters improved. During consolidation, anticoagulation was transitioned to rivaroxaban and temporarily interrupted during profound thrombocytopenia. PE subsequently resolved without recurrent thrombosis, but this single outcome does not establish the optimal drug, dose, platelet threshold, or duration for other patients. The documented anticoagulation course lasted approximately 4.5 months.

### 3.4. Clinical implications

Three practical lessons follow. First, unexplained leukocytosis, anemia, thrombocytopenia, or abnormal cells in a patient with PE should trigger manual peripheral-smear review and urgent hematology consultation; thrombocytopenia should not be assumed to result solely from infection, consumption, or medication. Second, once APL is suspected, ATRA and hemostatic support should not be delayed while genetic confirmation is pending.^[1]^ Serial platelet count, prothrombin time, activated partial thromboplastin time, fibrinogen, D-dimer, and clinical examination for both bleeding and thrombosis are essential during the early phase. Third, treatment of confirmed PE should be individualized according to hemodynamic status, thrombus burden, active bleeding, platelet and fibrinogen trends, and transfusion support. New symptoms during induction warrant evaluation for both hemorrhage and new thrombosis, even when a preexisting clot is radiologically improving.

### 3.5. Strengths and limitations

Strengths include definitive morphologic, cytogenetic, and molecular confirmation of APL; a time-stamped clinical course; serial imaging of both PE and splenic infarction; and molecular follow-up extending beyond 2.5 years. The focused literature review placed the case within the small body of comparable reports.

Several limitations require caution. A single case cannot estimate incidence, establish causality, or define a general anticoagulation algorithm. This report does not include every admission variable needed to verify a formal PE risk score, every serial hemostatic value, the anti-Xa unit concentration corresponding to the reported nadroparin syringe volumes, or all drug doses and transfusion thresholds; thus, some treatment decisions cannot be reproduced at a protocol level. The report also does not include mechanistic biomarkers or a uniform evaluation of every competing thrombotic cause, so the relative contributions of APL, conventional risk factors, dose-reduced anticoagulation, and differentiation therapy cannot be separated. We mitigated these limitations by distinguishing temporal association from causation, using serial objective imaging and molecular endpoints to describe the course, and avoiding a general treatment recommendation from a single case. Finally, the literature review was focused on PubMed and case reports rather than being systematic, making it susceptible to missed reports and publication bias; its findings provide clinical context, not a pooled estimate.

## 4. Conclusion

PE may precede recognition of APL and can coexist with severe thrombocytopenia and bleeding. PE accompanied by otherwise unexplained blood-count abnormalities should prompt immediate peripheral-smear review and evaluation for APL; suspected APL requires urgent ATRA and hemostatic support. When thrombosis is objectively confirmed, anticoagulation should be adjusted dynamically to thrombotic severity, active bleeding, and serial hemostatic parameters through multidisciplinary reassessment.

## Acknowledgments

The authors thank the patient for permitting publication of this report.

## Author contributions

**Data curation:** Jiayao Dong.

**Formal analysis:** Xiao Zhai.

**Writing – original draft:** Xiao Zhai.

**Writing – review & editing:** Xiao Zhai.
